# Pesticide Residues in Eggplant Fruit from Khartoum State, Sudan

**DOI:** 10.5696/2156-9614-10.25.200304

**Published:** 2020-02-28

**Authors:** Azhari Omer Abdelbagi, Rihab Eltahir Abdalla Ismail, Abd Elaziz Sulieman Ahmed Ishag, Ahmed Mohammed Ali Hammad

**Affiliations:** 1 Department of Crop Protection, Faculty of Agriculture, University of Khartoum, Khartoum, Sudan; 2 Ministry of Agriculture, Khartoum, Sudan

**Keywords:** eggplant, pesticide residues, Sudan

## Abstract

**Background.:**

Eggplant is a popular food item in Sudan, however pesticides are heavily used.

**Objective.:**

To investigate the presence of pesticide residues in fresh eggplants in Khartoum State, Sudan.

**Methods.:**

Eggplant fruit samples from three different regions in Khartoum State (central vegetable market, east Nile farms, and west Nile farms) were analyzed for residues of commonly used pesticides. Pesticide residues were analyzed by gas chromatography coupled with mass spectrometry and results were expressed in μg/kg fruit.

**Results.:**

Out of the 11 active ingredients analyzed, residues were identified for four pesticides (imidacloprid, dimethoate, endosulfan (α and β isomers) and 2, 4-D). Levels of omethoate, diazinon, malathion, chlorpyrifos, atrazine, and pendimethalin were below the detection limits.

**Conclusions.:**

Residues of four insecticides out of the 11 analyzed (imidacloprid, dimethoate, endosulfan (α, β isomers), and 2, 4-D) were detected in the current study. The health implications of these violative levels should be regularly observed along with strict enforcement of laws and regulations coupled with agricultural extension interventions.

**Competing Interests.:**

The authors declare no competing financial interests.

## Introduction

Vegetables are an important part of the human diet due to their high nutritional value and as important sources of vitamins (C, A, B6, thiamine, niacin, E), minerals, and dietary fiber.^1^,^2^ Production of vegetables in Sudan is rapidly increasing to meet the needs of the growing population. A wide range of vegetables and fruits are grown in Sudan due to the high availability of arable land, supply of irrigation water and suitable climatic conditions. According to the Food and Agriculture Organization of the United Nations (FAO), vegetable production in Sudan has increased annually in cultivated area and quantity.^3^ The percentage increases in cultivated area (hectares) and production (tons) between 2012–2017 were 31.67% and 86.78% for eggplant, 73.48% and 81.41% for tomatoes, 10.32% and 11.75 % for cucumbers, 78.48 and 88.44% for okra, 16.37% and 31.86 % for carrots, 61.74 % and 58.33% for onions, 26.10% and 28.53% for green chilies and peppers, and 68.92% and 71.38% for dry chilies and peppers, respectively. The main vegetables grown in the Sudan include tomato (Lycopersicom esculentum Miller), onion (Allium cepa), eggplant (Solanum melongena L.), and cucurbits.^4^

Eggplant, S. melongena L., is a typical and very profitable vegetable crop for farmers. It is locally known as bedingan or aswad and has several common names: aubergine, garden egg and brinjial.^5^,^6^ Eggplant come in different types, shapes, sizes, and colors.^7^ The popularity of eggplant in Sudan is mainly attributed to its affordability, the diversity of ways in which it can be cooked and its successful growth under warm weather conditions such as those in Sudan.^8^,^9^ The history of eggplant in Sudan is unknown, although its entry into Sudan was probably through Egypt.^10^ In 2017, the total area grown with eggplant in Sudan was about 13 000 hectares with a total production of 90 336 tons.^3^ Worldwide, eggplant production has been steadily increasing, with the largest production in China, India, Egypt, Turkey and Japan.^11^

However, eggplant production negatively affects the environment due to its heavy requirement for pesticides to combat insect pests and diseases. Insecticides are repeatedly applied during the entire period of growth and sometimes even at the fruiting stage. Indiscriminate use of pesticides during its farming season, particularly at the fruiting stage, and non-adoption of a pre-harvest interval may lead to accumulation of pesticide residues in the fruits, consumers and the environment.^12–15^

Previous studies indicate limited knowledge of vegetable farmers in Khartoum and Gezira states on the safe use of pesticides and their potential risks to human health and the environment.^16–18^ Extension services available to farmers were sub-standard and require more focus in current and future development projects. Furthermore, studies in Sudan indicate the presence of high levels of malathion, fenitrothion, chlorpyrifos, profenofos, dimethoate, heptachlor, diazinon, ethephon, oxyfluorfen and omethoate in fresh fruits.^17–23^ Prewashing fruits reduces the levels of some pesticides.^21^,^22^,^24^ Due to limited knowledge of pesticide safety, farmers indiscriminately and heavily use pesticides on eggplants. This study was initiated to investigate the presence of pesticide residues in fresh eggplant fruits in Khartoum State.

## Methods

**Figure 1:**
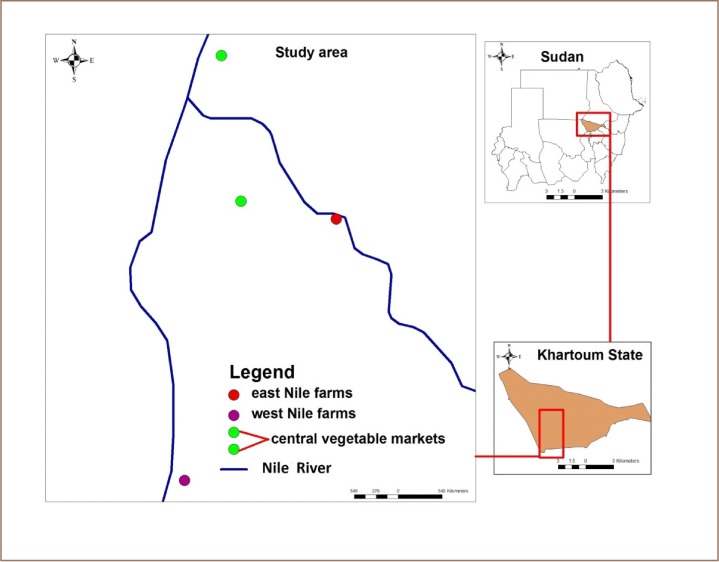
Sampling sites

Eggplant fruit samples (2–3 kg per sample) were collected randomly from three different areas in Khartoum State, Sudan: two central vegetable market locations (15.6726 N, 32.5376 E, 15.5304 N, 32.5576 E) and three east Nile farms (15.5133 N, 32.6534 E) and three west Nile farms (15.2578 N, 32.5015 E) *(Figure 1).* Twenty-seven (27) samples were collected in total, nine from each of the three study areas. The FAO Codex method was followed in sample collection at all locations.^25^ The collected samples were placed in paper bags, labeled and transferred immediately for extraction to the pesticide analytical laboratory, Faculty of Agriculture, University of Khartoum.

**Abbreviations**
*FAO*: Food and Agriculture Organization of the United Nations
*MRLs*: Maximum residue limits

### Chemicals and reagents

Ethyl acetate (purity 99.8%), acetone (purity 99.98%), anhydrous sodium sulphate (purity 99.5%), and dichloromethane (purity 99.98%) were purchased from El Waldain Company, Khartoum Arabic market. Analytical standards (99% purity) of endosulfan (α and β), dimethoate, diazinon, malathion, atrazine, chlorpyrifos, pendimethalin, omethoate, and imidacloprid were obtained from the Plant Protection Directorate (Ministry of Agriculture, Khartoum North, Sudan).

### Extraction and partitioning

The collected samples (unpeeled eggplant fruits) were sliced into small pieces, mixed thoroughly and about 50 g of each were weighed using a sensitive balance and blended with 10 g of anhydrous sodium sulphate at high speed (22000 rpm) for two minutes using a chemical resistent blender (National Analytical Corporation, Mumbai, India). The sample was then extracted with 100 ml acetone on a mechanical shaker for 1 hour using the method of Kumari *et al.*^26^ The extract was then filtered on Whatman filter No. 1, concentrated to 40 ml by rotary evaporator and subject to liquid-liquid extraction with ethyl acetate (50, 30, 20 ml) after dilutions with 100 ml 10% aqueous sodium chloride solution. The organic phase was collected in an Erlenmeyer flask and concentrated to 10 ml by rotary evaporator.

### Clean-up

Clean-up was carried out using a solid phase extraction column using ready-packed columns with silica gel and activated charcoal (5:1 wt/wt) topped with anhydrous sodium sulphate. The columns were pre-washed with 50 ml of acetone and hexane, respectively. Finally, the extract was passed through the columns and eluted with a 125 ml mixture of acetone:hexane (3:7 vol/vol). The cleaned extract was dried by rotary evaporator at 40°C until complete dryness and then reconstituted in 10 ml hexane and stored 4°C for analysis by gas chromatography coupled with mass spectrometry.

### Chromatographic analysis

**Figure 2:**
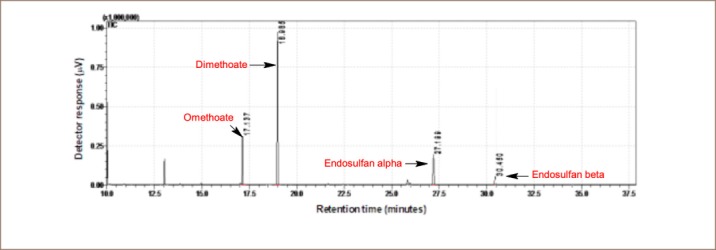
Chromatogram of omethoate, dimethoate, endosulfan alpha and endosulfan beta

**Figure 3:**
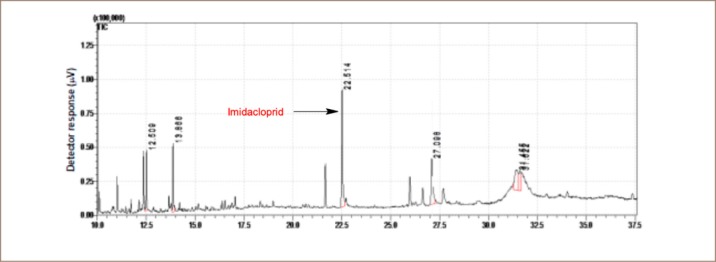
Chromatogram of imidacloprid

**Figure 4:**
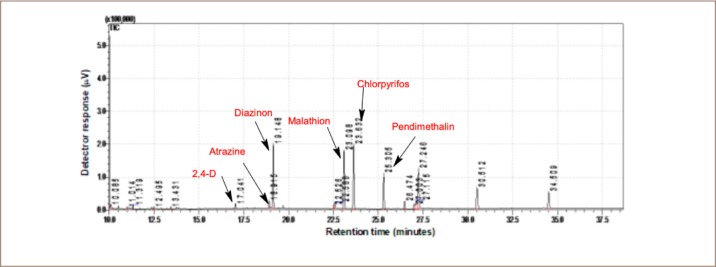
Chromatogram of 2, 4-D, atrazine, diazinon, malathion, chlorpyrifos and pendimethalin

The pesticide residue analysis was determined using gas chromatography coupled with mass spectrometry. Samples were analyzed using a Shimadzu GC-MSQP 2010 (Tokyo, Japan) with an AOC-5000 auto sampler. The gas chromatograph was fitted with an Rtx5-MS capillary column 30 m × 0.25 mm internal diameter × 0.25 μm film thicknesses from Restek (UK). Helium (purity ≥ 99.999%) was used as a carrier gas at a flow rate of 1.69 ml/min. The splitless injection temperature was 230°C. The oven temperature was programmed from initial temperature of 50°C for 3 minutes, raised at 10°C per minute to 200°C and held for 5 minutes, then increased by 3°C per minute to 230°C and held for 2 minutes. The mass spectrometer was operated with an electron impact source in scan mode. The electron energy was 70 eV, and the interface temperature was maintained at 200°C. The solvent delay was set to 10 minutes. The retention time of standard pesticides are shown in Figures 2, 3 and 4. Detection limits are given in Table 1. The recovery of the method ranged from 70–120%. No pesticide residues were detected in the calibration blanks.

**Table 1:** Minimum Detectable Levels and Retention Times of Studied Pesticides

**Pesticides**	**CAS number^27^,^28^**	**Minimum detectable level (μg/kg)**	**Retention time (minutes)**
2,4-D	94-75-7	30	17.029
Omethoate	1113-02-6	50	17.137
Dimethoate	60-51-5	30	18.904
Atrazine	1912-24-9	10	19.153
Diazinon	333-41-5	50	19.739
Imidacloprid	138261-41-3	50	22.514
Malathion	121-75-5	50	23.089
Chlorpyrifos	2921-88-2	50	23.622
Pendimethalin	40487-42-1	10	25.297
Endosulfan α	115-29-7	10	27.230
Endosulfan β	115-29-7	10	30.450

Abbreviation: CAS number, Chemical Abstract Service Registry Number.

The pesticide residues present in the eggplant samples were identified by matching their retention times and mass-spectrum to those of analytical standards. About 1.0 μl of various concentrations of each analytical standard was injected in the gas chromatography and their peak areas were used for the construction of the standard curves. Duplicate samples of 1 μl from each extract were injected and concentration was determined using Equation 1.

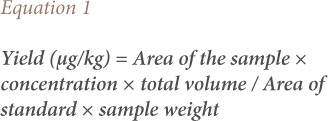



## Results

The results of residues analysis of samples are summarized in Tables 2 and 3 and Supplemental Material. Residues were detected in 11% of samples in central vegetable market, 33% of samples from east Nile farms and 22% of samples of west Nile farms. Average violative levels (> FAO Codex maximum residue limits (MRLs)) ranged from 11% (endosulfan α) to 55% (imidacloprid). The highest violations were found to be associated with east Nile farms samples, while the lowest were found to be associated with central vegetable market samples. The most frequently detected pesticides were imidacloprid, dimethoate and β endosulfan (in 66% of samples), followed by 2,4-D (in 33% of samples), and α endosulfan (in 22% of samples). The highest detected levels were found to be associated with the insecticide imidacloprid in all samples, with an average of 1650 μgkg^−1^ in central vegetable market samples, 2640 μgkg^−1^ in east Nile farms samples, and 1547 μgkg^−1^ in west Nile farms samples with corresponding ranges of ND-4960 μgkg^−1^, 200–4760 μgkg^−1^ and ND-2957 μgkg^−1^.

**Table 2:** Levels of Insecticides Residues (μgkg^−1^) Detected in Eggplant Samples Collected from Central Vegetable Market and Vegetable Farms in Khartoum State

**Insecticides**	**Levels**	**Central vegetable market**	**East Nile farms**	**West Nile farms**
α Endosulfan	Average	ND	1040	ND
	Range	ND	ND-3050	ND
	Median	ND	50	ND
	No. of sample tested + ve(%)	ND	66	ND
	SET	ND	101	ND
	Violative (%) sample^*^	ND	33	ND
β Endosulfan	Average	50	418	70
	Range	ND-140	164–1090	ND-120
	Median	-	-	366
	No. of sample tested + ve(%)	33	100	66
	SE±	465	108	365
	Violative (%) sample^*^	ND	ND	ND
Total endosulfan		50	1458	70
Omethoate	Average	ND	ND	ND
	Range	ND	ND	ND
	Median	ND	ND	ND
	No. of sample tested + ve(%)	ND	ND	ND
	SE±	ND	ND	ND
	Violative (%) sample^*^	ND	ND	ND
Dimethoate	Average	427	77.3	94.1
	Range	ND-1280	51.1–1042	ND-178.2
	Median	200	76.4	104.3
	No. of sample tested + ve(%)	33	100	66
	SE±	170	15.3	51.7
	Violative (%) sample^*^	ND	ND	ND
Diazinon	Average	ND	ND	ND
	Range	ND	ND	ND
	Median	ND	ND	ND
	No. of sample tested + ve(%)	ND	ND	ND
	SET	ND	ND	ND
	Violative (%) sample^*^	ND	ND	ND
Malathion	Average	ND	ND	ND
	Range	ND	ND	ND
	Median	ND	ND	ND
	No. of sample tested + ve(%)	ND	ND	ND
	SE±	ND	ND	ND
	Violative (%) sample^*^	ND	ND	ND
Chlorpyrifos	Average	ND	ND	ND
	Range	ND	ND	ND
	Median	ND	ND	ND
	No. of sample tested + ve(%)	ND	ND	ND
	SE±	ND	ND	ND
	Violative (%) sample^*^	ND	ND	ND
Imidacloprid	Average	1650	2640	1547
	Range	ND-4960	200–4760	ND-2957
	Median	-	2970	1683
	No. of sample tested + ve(%)	33	100	66
	SET	1650	1330	856
	Violative (%) sample^*^	33	66	66
Insecticides load/kg		1742.7	4175.3	1711.1

Note: Average load/kg = 7629.1 μg/kg; Grand range = ND-4957 μg/kg; Total of samples tested + ve (%) = 27.

Abbreviations: +ve, positive; SE, standard error.

^*^Percentage of samples with level exceeding the MRLs cited by FAO and World Health Organization^29^

**Table 3:** Pesticides Detected in Eggplant Samples Collected from Three Study Areas in Khartoum State and Vegetable Farms in Khartoum State

**Study area**	**Pesticides**	**Retention time (minutes)**
**East Nile farms**	2,4-D	17.029
	Dimethoate	18.904
	Imidacloprid	22.514
	Endosulfan α	27.230
	Endosulfan β	30.450
**Central vegetable market**	Dimethoate	18.904
	Imidacloprid	22.514
	Endosulfan β	30.450
**West Nile farms**	2,4-D	17.029
	Dimethoate	18.904
	Imidacloprid	22.514
	Endosulfan β	30.450

Generally, endosulfan α showed the lowest levels, except in east Nile farm samples where its average was 1040 μgkg^−1^ with a range of ND-3050 μgkg^−1^. The highest insecticide loads per kilogram fruit were found in the samples collected from east Nile farms (average total 4175.3 μgkg^−1^), followed by central vegetable market (1742.7 μgkg^−1^) and west Nile farms (1711.1 μgkg^−1^). In addition, the highest frequency of detection (+ ve samples %) and the highest frequency of violative levels (violation %) followed the same order *(Tables 2 and 3 and Supplemental Material).* Levels of omethoate, diazinon, malathion, chlorpyrifos, atrazine, and pendimethalin were below the detection limit *(Tables 2 and 4).*

**Table 4:** Levels of Herbicides Residues (μgkg^−1^) Detected in Eggplant Samples Collected from Central Vegetable Market and Vegetable Farms in Khartoum State

**Herbicides**	**Levels**	**Central vegetable market**	**East Nile farm**	**West Nile farm**
Atrazine	Average	ND	ND	ND
	Range	ND	ND	ND
	Median	ND	ND	ND
	No. of sample tested + ve(%)	ND	ND	ND
	SE±	ND	ND	ND
	Violative (%) sample^*^	ND	ND	ND
Pendimetnahn	Average	ND	ND	ND
	Range	ND	ND	ND
	Median	ND	ND	ND
	No. of sample tested + ve(%)	ND	ND	ND
	SE±	ND	ND	ND
	Violative (%) sample^*^	ND	ND	ND
2,4-D	Average	ND	38.7	31.1
	Range	ND	ND-133.49	ND-113
	Median	ND	102.8	-
	No. of sample tested + ve(%)	ND	66	33
	SE±	ND	40.4	37
	Violative (%) sample^*^	ND	ND	ND
Total of herbicides		ND	38.7	37.7

Note: Average load/kg = 38.2 μ/kg; Grand range = ND-133.49 μ/kg; Total number samples tested +ve(%) = 11.

Abbreviations: ND, not detected; No., number; +ve, positive; SE, standard error.

^*^Percentage of samples with level exceeding the MRLs cited by FAO and World Health Organization.^29^,^27^

The herbicide 2,4-D was the only herbicide detected and only in samples from east Nile and west Nile farms. Its level was relatively higher in east Nile farms (38.7 μgkg^−1^) versus west Nile farm samples (37.7 μgkg^−1^). Its frequency of detection followed the same order (66% in east Nile farms versus 33% in west Nile farms). Herbicide 2,4-D levels detected were below FAO Codex MRLs. Atrazine and pendimethalin levels were below the detection limit *(Table 4 and Supplemental Material).*

In summary, the average residue load of insecticides and herbicides per kg fruit was about 2443.1 μgkg^−1^ with a load range of ND-3892.7 μgkg^−1^, while the total frequency of detection of pesticide residues was 66% *(Table 5).* Out of the total samples, about 55% were violative. Generally, the highest levels were detected in the east Nile farm samples (3837.8 μgkg^−1^) followed by west Nile farm samples (1748.8 μgkg^−1^) and central vegetable market samples (1742.7 μgkg^−1^). In addition, the level of violations and the frequency of detection followed the same order *(Table 3,5 and Supplemental Material).*

**Table 5:** Summary of Pesticides Residue (μgkg^−1^) Detected in Eggplant Samples Collected from Central Vegetable Market and Vegetable Farms in Khartoum State

**Locations (Coordinates)**	**Pesticide group**	**Total average**	**Total range**	**Violative (%)**
Central vegetable market (15.6726 N, 32.5376 E, 15.5304 N, 32.5576 E)	Insecticides	1742.7	ND-4960	33
	Herbicides	ND	ND	ND
	Total	1742.7	ND-4960	33
East Nile farms (15.5133 N, 32.6534 E)	Insecticides	3799.1	ND-4760	66
	Herbicides	38.7	ND-134	ND
	Total	3837.8	ND-4894	66
West Nile farms (15.2578 N, 32.5015 E)	Insecticides	1711.1	ND-1711.1	66
	Herbicides	37.7	ND-113	ND
	Total	1748.8	ND-1824.1	66

Note: Average load/kg fruit = 2443.1 μg/kg; Average range of the load/kg fruit = ND-3892.7 μg/kg; Total of violations in sample (%) = 55.

Abbreviation: ND, not detected.

## Discussion

The current study investigated the level of 11 commonly used pesticides in eggplant samples collected from vegetable farms and central vegetable market in Khartoum State, Sudan. Detectable levels were associated with only four pesticides (imidacloprid, dimethoate, endosulfan (α, β isomers) and 2,4-D). One (1) or more of these pesticides were found in many samples from all locations. Not all detected pesticides are authorized for use on eggplant in Sudan (A.H. Ahmed, Plant Protection Directorate, personal communication September 2019).

However, out of 11 studied pesticides, only two were insecticides authorized for use on eggplant in the Sudan: omethoate and dimethoate. The rest are either registered for use on other vegetables or not registered for use on crops. These were included in the present study as farmers interviewed in these areas reported their use on crops.^30^ This reflects the urgent need for extension services to be available for farmers in these areas. The current results are in line with results obtained by Hammad *et al.* who found that tomato samples collected from greenhouses in different locations in Khartoum state contained lambda-cyhalothrin and imidacloprid residues.^20^ Levels detected in their study were higher than MRLs established by either Codex Alimentarius or the European Union (EU).^3^,^31^ Contrary to the current result, Ahmed *et al.* reported that imidacloprid and its metabolite residues were below the detection limit (0.09 μg) in all samples analyzed (dates, soil and intercropped plants (alfalfa, and grasses) even at the highest dose applied (35 ml/palm).^32^ Similar to the current results, Daraghmeh *et al.* found imidacloprid residues in more than half of the analyzed samples in the West Bank, Palestine, although levels detected were lower than those reported in the current study.^33^ The highest level of imidacloprid reported in the study was found in eggplant (460 μgkg^−1^), while the lowest level was found in green beans (80 μgkg^−1^). An increase (11–120%) in imidacloprid concentration was observed in samples from 1999 compared to samples from 1998. Daraghmeh *et al.* attributed this increase to a possible accumulation of imidacloprid residues in the soil and/or to increased use by local farmers.

Dimethoate residues were detected in 33% of samples from central vegetable market, 100% of samples from east Nile farms and 66% of samples from west Nile farms. Its respective average concentrations (μgkg^−1^) and ranges (μgkg^−1^) were 42.7, 77.3 and 94.1; ND-1280, 51.1–1042 and ND-178.2. The current results partially agree with those of Aldawi *et al.* and Musa *et al.* who found higher levels of dimethoate residues (exceeding EU MRLs) in cucumber and sweet pepper samples collected from Khartoum State, Sudan.^21^,^22^ In contrast, lower levels (0.22 μgkg^−1^) of dimethoate residues were reported in eggplant samples collected from Ghanaian markets.^34^ The variation across these studies may be explained by differences in location and use patterns in different vegetable crops.

Residues of endosulfan β were detected in 33% of the samples collected from central vegetable market, 100% of the samples collected from east Nile farms and 66% of the samples collected from west Nile farms. The current results partially agree with Aldawi *et al.* who found residues of endosulfan α in some samples of cucumbers fruit collected from open fields in Khartoum State, Sudan at a frequency of detection ranging from below the detection limit to 33.3%.^21^ Residues of endosulfan β were not detected in the samples analyzed.^21^ On the other hand, Thanki *et al.* found residues of endosulfan β ranging from 1280–1420 μgkg^−1^ in row eggplant samples from Gujarat, India.^11^ In contrast, lower levels (<0.01 μgkg) of endosulfan residues were reported in eggplant samples collected from Ghanaian markets.^34^ Variations across these studies may be explained by differences in location and pesticide use patterns in different vegetable crops.

Endosulfan is the only organochlorine pesticide still permitted for agricultural use in Sudan, where it previously constituted at least 50% of the annual spray regime in cotton until 1992.^35^ Cotton plant shoots, soil and canal water were sampled and analyzed for residues of endosulfan sprayed on cotton during 1982–1983. Detectable residues were found in all three media, one month after spraying. Concentrations of 590 μgkg^−1^, 2550 μgkg^−1^ and 2789 μgkg^−1^ were detected in soil, water and plants, respectively.^36^ The occurrence of endosulfan in the samples may be explained by a possible recent illegal use on eggplants, as this insecticide is not registered for vegetable use. Farmers in the area claim to use endosulfan for pest control.^30^ However, endosulfan does not have a long environmental persistence as a parent compound, but instead it forms a persistent metabolite, endosulfan sulfate.^27^ Previous studies found endosulfan residues in water, soil, blood and food items in the Sudan.^12–14^,^37^

Furthermore, endosulfan was reported as the main causative agent of human poisoning in Sudan,^38–40^ although most of the reported cases were due to consumption of endosulfan-contaminated food.

The herbicide 2,4-D was detected in samples from east Nile and west Nile farms, with a relatively higher level in the east Nile farms. The presence of 2,4-D in the samples may be due to its recent use in the area (although not claimed by the farmers interviewed), or from drift from nearby farms or from contaminated equipment.^30^ Fantke *et al.* reported that 2,4-D and parathion were the most damaging pesticides across the various crops studied.^41^

Levels of omethoate, diazinon, malathion, chlorpyrifos, atrazine and pendimethalin were below the detection limit. The absence of their residues may be explained by the absence or limited use in the area. Previous studies in Sudan indicated the presence of high levels of malathion, fenitrothion, chlorpyrifos, profenofos, dimethoate, heptachlor, diazinon, ethephon, oxyfluorfen, dimethoate and omethoate in fresh vegetables.^16–21^ Variation across studies may be explained by differences in location and use patterns in different vegetable crops.

Violations (greater than FAO-Codex MRLs) ranged from 11% (endosulfan α) to 55% (imidacloprid). The highest violations were associated with east Nile farms, while the lowest were associated with central vegetable market samples. This partially agrees with Musa *et al.* and Aldawi *et al.* who reported the highest frequency (100%) of violations (>MRLs) corresponding to dimethoate, endosulfan β, omethoate, dimethoate and heptachlor.^21^,^22^ The highest levels, detection and violation frequencies were associated with east Nile farms, while the lowest were associated with central vegetable market samples. The results confirm the presence of residues of some of the most commonly used pesticides in the study area at violative levels, indicating the need for a regular residue monitoring program in these crops. The limited knowledge of farmers on the safe use of pesticides and limited training programs available indicate the need for immediate intervention and enforcement of safety limits. As violative levels were found for imidacloprid and endosulfan, immediate review of their use in vegetables in Sudan is needed, along with detailed studies about the potential health effects which may occur from the consumption of contaminated fruits. Furthermore, strict regulations should be enforced to prevent illegal use of endosulfan in vegetables. Provision of extension services and information on the safe use of pesticides to vegetable farmers are needed to mitigate potential risks to human health and the environment.

Limitations of the present study included a lack of detailed information on pesticide use patterns and safety aspects as well as lack of policy enforcement in the study areas. In addition, the effect that processing eggplant fruits has on the level of pesticide residues was not considered. Further studies examining pesticide residues in other commonly consumed vegetables in the Sudan are needed as there is no regular monitoring program for pesticide residues in vegetables in this area and few studies have examined this issue.

## Conclusions

Residues of four insecticides out of the 11 analyzed (imidacloprid, dimethoate, endosulfan (α, β isomers), and 2, 4-D) were detected in the current study. Violative levels were found for imidacloprid and endosulfan. The highest levels and frequency of detection and violation were found for imidacloprid. The highest levels and frequency of detection and frequency of violation were associated with east Nile farms, while the lowest were associated with central vegetable market. Levels of omethoate, diazinon, malathion, chlorpyrifos, atrazine, pendimethalin were below detection limits. The health implications of these violative levels should be regularly observed along with strict enforcement of laws and regulations coupled with agricultural extension interventions.

## References

[1] Wargovich MJ (2000). Anticancer properties of fruits and vegetables. HortScience [Internet].

[2] Slavin JL, Lloyd B (2012). Health benefits of fruits and vegetables. Adv Nutr [Internet].

[3] (1961). FAOSTAT: crops [Internet].

[4] Abdalla AA (1966). Horticultural aspect of crop diversification in the Sudan.

[5] Hill DS (1975). Agricultural insect pests of the tropics and their control.

[6] Tindall HD (1983). Vegetables in the tropics.

[7] Yamaguchi M (1983). World vegetables: principles, production and nutritive values.

[8] Sampson AB (1997). Comparisons of three methods for establishing economic threshold levels for Jacobiasca lypica (de Berg) on eggplant [dissertation].

[9] Elkhaliefa SH (1999). Studies on the biology and ecology of the eggplant fruit-borer Daraba Laisalis (walk) (Lepidoptera: pyralidae) [master's thesis].

[10] Osman ME (1984). Evaluation of eggplant cultivars in the Sudan. Acta Hortic.

[11] Oluoch MO, Chadha ML (2007). Evaluation of African eggplant for yield and quality characteristics. Acta Hortic.

[12] Thanki N, Joshi P, Joshi H (2012). Effect of household processing on reduction of pesticide residues in Bringal (eggplant, Solanum melongena). Adv Appl Sci Res.

[13] Nesser GA, Abdelbagi AO, Hammad AM, Tagelseed M, Laing MD (2016). Levels of pesticides residues in the White Nile water in the Sudan. Environ Monit Assess [Internet].

[14] Elbashir AB, Abdelbagi AO, Hammad AM, Elzorgani GA, Laing MD (2015). Levels of organochlorine pesticides in the blood of people living in areas of intensive pesticide use in Sudan. Environ Monit Assess [Internet].

[15] Abdelbagi AO, Elbashir AB, Hammad AM, Elzorgani GA, Laing MD (2015). Organochlorine levels in human blood from residents in areas of limited pesticide use in Sudan. Toxicol Environ Chem [Internet].

[16] Ahmed RA (2002). Agricultural extension role to reduce the pesticides exposure hazards and their effect: case study, Gezera scheme's vegetable farmers.

[17] Mohamed AO, Mater AA, Hammad AM, Ishag AE, Abdelbagi AO, Eldein AM, Eltayeb EM, Dahab AA, Gader AA (2019). Knowledge, attitudes and practices of pesticide's sprayers towards pesticides use and handling in greenhouse farms, Sudan. Int J Manag Commer Innov.

[18] Mohamed AO, Mater AA, Hammad AM, Ishag AE, Eldein AM, Eltayeb EM, Dahab AA, Gader AA, Abdelbagi AO (2018). Knowledge, attitudes and practices of farmers towards pesticides use and handling in greenhouse farms, Sudan. Int J Res Granthaalayah.

[19] Hammad AM, Abdelbagi AO, Ishag AE Ahmed A, Laing MD Determination of residues levels of seven pesticides in tomatoes samples taken from three markets in Khartoum State, Sudan.

[20] Hammad AM, Yasein BH, Ishag AE, Abdelbagi AO, Laing MD (2015). Detection of insecticide residues on tomato fruits grown in greenhouses in Khartoum State. Univ Khartoum J Agric Sci.

[21] Aldawi MM, Abdelbagi AO, Ishag AE, Hammad AM (2019). The level of pesticide residues in cucumber fruits collected from central vegetable markets in Khartoum State. EC Pharmacol Toxicol.

[22] Musa NH, Hammad AM, Abdelbagi AO, Ishag AE (2019). Pesticides residues in samples of sweet peppers (Capsicum annum) from Khartoum State, Sudan. EC Pharmacol Toxicol.

[23] Mohamed AO, Mater AA, Hammad AM, Ishag AE, El Tayeb EM, Dahab AA (2018). Pesticide residues detected on tomato and cucumber fruits grown in greenhouse farms in Khartoum State, Sudan. Int J Life Sci Res.

[24] Abdalla KM (2005). Evaluation of field performance of selected insecticides against major pests of tomatoes, associated residues in fruits and farmers knowledge about their use [master's thesis].

[25] (1995). Codex general standard for contaminants and toxins in food and feed: CODEX STAN 193-1995 [Internet].

[26] Kumari B, Madan VK, Kumar R, Kathpal TS (2002). Monitoring of seasonal vegetables for pesticide residues. Environ Monit Assess [Internet].

[27] (c2020). VO 0440 - Egg plant [cited 2020 Feb 11]. Codex Pesticides Residues in Food Online Database [Internet].

[28] Fantke P, Arnot JA, Doucette WJ (2016). Improving plant bioaccumulation science through consistent reporting of experimental data. J Environ Manage [Internet].

[29] (c2020). Codex Pesticides Residues in Food Online Database [Internet].

[30] Ismail RE (2016). Pesticide residues in eggplant (and evaluation of farmers knowledge about proper use of pesticides in Khartoum State) [master's thesis].

[31] Regulation (EC) No. 396/2005 of the European Parliament and of the Council of 23 February 2005 on maximum residue levels of pesticides in or on food and feed of plant and animal origin and amending Council Directive 91/414/EEC.

[32] Ahmed MA, Abdelbagi AO, Elshafie HA, Fageer EA, Abass IA (2013). Efficacy of Imidacloprid (Confidor 200SL) and improved cultural practices in the control of the green date palm pit scale insect (Asterolecanium phoenicis Rao.) (Palmapsis phoenicis) (Homoptera: Asterolecaniidae) in northern Sudan. Sci Res Essays.

[33] Daraghmeh A, Shraim A, Abulhaj S, Sansour R, Ng JC (2007). Imidacloprid residues in fruits, vegetables and water Samples from Palestine. Environ Geochem Health.

[34] Botwe BO, Ntow WJ, Kelderman P, Drechsel P, Carboo D, Nartey VK, Gijzen H J (2011). Pesticide residues contamination of vegetables and their public health implications in Ghana. J Environ Issues Agric Dev Ctries.

[35] Abdelbagi AO (2006). Pesticide use and management in the Sudan. National workshop on insecticide resistance and its management.

[36] Abdalla AT, Satti AM, Yousif G, Moghraby AI (1985). Residues of the organochlorine insecticide endosulfan, in the Gezira Scheme, Sudan. Sudan J Sci.

[37] Assad YO, Bashir HN (2015). Persistent toxic substances (PTS) in the Sudan.

[38] Diarra L, Kamissoko M, Alsaffar A, Eldin SM (1992). IPM and pesticide use in Mali and Sudan.

[39] Alhindi AM (1994). Food contamination, pesticide poisoning episodes and methods of sampling. Training course on the use of pesticides (in Arabic).

[40] Abdelbagi AO (2005). Assessment of national POPs monitoring and research capacity for persistent organic pollutants (POPs) in the Sudan, their levels in the Sudanese environment, human and animal exposure.

[41] Fantke P, Friedrich R, Jolliet O (2012). Health impact and damage cost assessment of pesticides in Europe. Environ Int [Internet].

