# Supplementary material for: Pesticide Residues in Eggplant Fruit from Khartoum State, Sudan
Source: J Health Pollut. 2020 Feb 28;10(25):200304. doi: 10.5696/2156-9614-10.25.200304 (PMC7058141; doi:10.5696/2156-9614-10.25.200304)
Supplement: Supplementary file 1 [file Abdelbagi_Supplemental.docx]

**Supplemental Material**

Typical chromatogram of 2, 4-D, dimethoate, imidacloprid and β endosulfan pesticides detected in east Nile farm samples

Typical chromatogram of dimethoate, imidacloprid, α endosulfan and β endosulfan insecticides detected in east Nile farm samples

Typical chromatogram of 2, 4-D, dimethoate, imidacloprid and β endosulfan pesticides detected in east Nile farm samples

Typical chromatogram of dimethoate, imidacloprid and β endosulfan insecticides detected in central vegetable market samples

Typical chromatogram of 2, 4-D, dimethoate, imidacloprid and β endosulfan pesticides detected in west Nile farm samples
